# The Symbiotic “All-Rounders”: Partnerships between Marine Animals and Chemosynthetic Nitrogen-Fixing Bacteria

**DOI:** 10.1128/AEM.02129-20

**Published:** 2021-02-12

**Authors:** Jillian M. Petersen, Benedict Yuen

**Affiliations:** a Centre for Microbiology and Environmental Systems Science, University of Vienna, Vienna, Austria; University of Tennessee at Knoxville

**Keywords:** nitrogen fixation, symbiosis

## Abstract

Nitrogen fixation is a widespread metabolic trait in certain types of microorganisms called diazotrophs. Bioavailable nitrogen is limited in various habitats on land and in the sea and, accordingly, a range of plant, animal, and single-celled eukaryotes have evolved symbioses with diverse diazotrophic bacteria, with enormous economic and ecological benefits. Until recently, all known nitrogen-fixing symbionts were heterotrophs, such as nodulating rhizobia, or photoautotrophs, such as cyanobacteria. In 2016, the first chemoautotrophic nitrogen-fixing symbionts were discovered in a common family of marine clams, the Lucinidae. Chemosynthetic nitrogen-fixing symbionts use the chemical energy stored in reduced sulfur compounds to power carbon and nitrogen fixation, making them metabolic “all-rounders” with multiple functions in the symbiosis. This distinguishes them from heterotrophic symbionts that require a source of carbon from their host, and their chemosynthetic metabolism distinguishes them from photoautotrophic symbionts that produce oxygen, a potent inhibitor of nitrogenase. In this review, we consider evolutionary aspects of this discovery, by comparing strategies that have evolved for hosting intracellular nitrogen-fixing symbionts in plants and animals. The symbiosis between lucinid clams and chemosynthetic nitrogen-fixing bacteria also has important ecological impacts, since they form a nested symbiosis with endangered marine seagrasses. Notably, nitrogen fixation by lucinid symbionts may help support seagrass health by providing a source of nitrogen in seagrass habitats. These discoveries were enabled by new techniques for understanding the activity of microbial populations in natural environments. However, an animal (or plant) host represents a diverse landscape of microbial niches due to its structural, chemical, immune, and behavioral properties. In the future, methods that resolve microbial activity at the single cell level will provide radical new insights into the regulation of nitrogen fixation in chemosynthetic symbionts, shedding new light on the evolution of nitrogen-fixing symbioses in contrasting hosts and environments.

## Abstract (precis)

Nitrogen fixation is a widespread metabolic trait in certain types of microorganisms called diazotrophs. Bioavailable nitrogen is limited in various habitats on land and in the sea and, accordingly, a range of plant, animal, and single-celled eukaryotes have evolved symbioses with diverse diazotrophic bacteria, with enormous economic and ecological benefits.

## INTRODUCTION

## DEFINING CHEMOSYNTHETIC SYMBIOSES

Chemosynthetic symbioses are classically defined as intimate associations between eukaryotic hosts and either of two types of bacterial symbionts: (i) chemolithoautotrophic bacteria, which fix inorganic carbon using the energy released by oxidation of reduced chemical compounds such as hydrogen sulfide, or (ii) methane-oxidizing bacteria, which synthesize their biomass from the single-carbon compound methane (1–3). Both of these symbiont types play the role of primary producers in the symbiosis, harnessing chemical energy to synthesize organic matter for their own and their host’s nutrition.

It is becoming clear that even this broad definition of chemosynthetic symbioses is deceptively straightforward. It encompasses a diverse assortment of associations that cover virtually all extremes of intimacy and reliance on the symbiotic lifestyle. For example, thyasirid bivalves host ectosymbiotic chemolithoautotrophic bacteria on gill epithelial cells, which regularly engulf and digest the ectosymbionts. Symbiont-hosting and symbiont-free individuals of one particular thyasirid species are found cooccurring in nature, which shows that this association is likely an optional nutritional supplement, rather than essential for survival (4–6). The symbionts have estimated genome sizes typical of their closest free-living relatives and can probably survive in a free-living form in the environment (7). Most chemosynthetic symbioses are horizontally transmitted, meaning they are acquired from the environment or from cooccurring hosts during development (8). This is consistent with the observation that vertical transmission, the direct transfer of symbionts from parent to offspring, is rare in marine symbioses between animals and bacteria (9). However, there are examples of vertically transmitted chemosynthetic symbioses. At the other extreme, a different bivalve group, the vesicomyids, host chemoautotrophic endosymbionts inside gill epithelial cells. These vertically transmitted endosymbionts have reduced genomes, a hallmark of obligate association with the host, and the bivalves concurrently have a reduced digestive tract, a hallmark of a long evolutionary history of relying on a symbiotic source of nutrition (10–12). Associations are also known that are absolutely essential to the survival of one partner but seem to be optional for the other, such as the hydrothermal vent tubeworm *Riftia pachyptila*, which lacks a digestive system, relying entirely on its intracellular symbionts for nutrition. In contrast to the host’s absolute dependency on the symbionts as adults, the symbionts can make a living by themselves in the surrounding environments and are acquired from the environment by juvenile hosts during development (13, 14). Despite these massive variations in the form and function of chemosynthetic symbioses, most are found in marine environments (see reference 15 for the only known exception), and although they evolved multiple times independently in numerous eukaryotic and bacterial groups, most currently known hosts of chemosynthetic symbioses are invertebrate animals, and most currently known symbionts are members of the *Gammaproteobacteria*. Other excellent reviews provide more details on the diversity, ecology, evolution, and transmission modes of chemosynthetic symbioses than is possible in this contribution (1, 2, 8, 16, 17).

## SYMBIONT METABOLISM: DIVERSITY OF ENERGY SOURCES AS A UNITING FEATURE

Despite almost 40 years of intensive study on chemosynthetic symbioses, the first and (thus far) only chemosynthetic symbiont was brought into pure culture only recently (18). Most efforts to cultivate these symbionts in the lab fail, even for those symbionts thought to have a free-living environmental stage. Because of this, studying these symbioses requires the concepts and technologies of environmental microbiology, the study of microbes in their natural environments. This may seem like a handicap, but it is thanks to this “limitation” that chemosynthetic symbioses are one of the best-developed experimental models for understanding host-microbe-environment interactions. Molecular methods from “omics” to imaging have transformed this field, revealing that there is virtually only one uniting feature of chemosynthetic symbionts, a diversity of metabolic capabilities. CO_2_ and CH_4_ were thought to be the only two carbon sources used by chemosynthetic symbionts, CO_2_ by chemolithoautotrophs, CH_4_ by methanotrophs. Genome sequencing of diverse symbionts is beginning to reveal that surprisingly, although considered archetypical chemolithoautotrophs, most sulfide-oxidizing symbionts have the potential for heterotrophic growth (19–24). This heterotrophic potential remains to be experimentally tested. One sulfide-oxidizing symbiont that completely lacks known pathways for autotrophy was recently discovered as an ectosymbiont on a single-celled protist host (25). Similar symbionts have not yet been found in animal hosts. The ability to fix inorganic carbon was possibly lost as the symbiont evolved to specialize in “toxic” waste recycling for its host, which inhabits anoxic marine habitats and produces inhibitory (“toxic”) organic compounds during anaerobic metabolism. H_2_S and CH_4_ were also the only known energy sources (electron donors) known to power chemosynthetic symbioses for the first 30 years of research on these associations, but this limited picture has now also been overturned with first the discovery of hydrogen-powered symbioses in the deep sea, as well as subsequent discoveries revealing an expanding range of energy sources that fuel chemosynthetic primary production, which include carbon monoxide and potentially also reduced iron (26–30).

## LUCINID CLAM SYMBIOSIS

Lucinidae is one of the most species-rich animal families in the oceans today, containing at least 400 described species (31). Each species thus far investigated hosts intracellular bacterial symbionts in epithelial cells of the gill. Our understanding of the overall biodiversity of the bacterial symbionts lags surprisingly far behind that of the hosts, whose evolutionary history has been the subject of intensive study (31–36). The few symbionts that have been sequenced mostly belong to a single, possibly family-level group of bacteria. They are related to cultured sulfide oxidizers of the genus *Sedimenticola* and, surprisingly, also the endosymbionts of deep-sea *Riftia* tubeworms (20, 21, 37). Molecular studies targeting the 16S rRNA gene using different methods such as direct Sanger sequencing of PCR products, clone library Sanger sequencing, and high-throughput amplicon sequencing concur that each host individual harbors a single dominant symbiont phylotype. More recent analyses, including high-throughput 16S rRNA gene sequencing, and other marker genes, revealed the possible presence of additional rare phylotypes in gill samples (32–34). Since many of these rare sequence types were consistently found across multiple host individuals, they likely represent true symbionts rather than sequencing errors, but imaging methods such as fluorescence *in situ* hybridization are still needed to show that multiple symbiont types cooccur. Therefore, despite enormous improvements in the resolution of these biodiversity assessments, the original observation that each host individual harbors a single dominant symbiont type remains valid.

Patterns of host-symbiont biodiversity and specificity are complex in lucinid symbioses. Individuals of different host species can harbor virtually identical symbiont types, particularly when these host species cooccur (38, 39). In other chemosynthetic symbioses, cooccurring host species host their own exclusive species-specific symbionts; thus, the lucinid symbioses are considered unusually flexible in the range of symbiont genotypes they can potentially form associations with (38, 40–42). At the same time, two cooccurring lucinid individuals of the same species can each harbor a distinct symbiont type, which, although almost identical at the 16S rRNA level, share as little as 83% nucleotide identity across their entire approximately 4- to 5-Mb genomes (20, 21). These would be considered distinct symbiont species, possibly even distinct genera (43). Symbionts with this much variability in genome sequence and content are very likely functionally distinct (30), but this remains to be investigated and tested experimentally. If so, this would mean that lucinids of the same species can thrive in the same habitat with different, functionally distinct symbionts. This flexibility is also unusual among chemosynthetic symbioses, where cooccurring host individuals of the same species tend to harbor the same symbiont type(s) (20, 21, 37).

## CONTEXT OF THE DISCOVERY OF NITROGEN-FIXING CHEMOSYNTHETIC SYMBIONTS

Soon after their key role as primary producers fixing inorganic carbon was discovered, vent researchers considered the possibility that chemosynthetic symbionts might also fix nitrogen (44–46). Indeed, nitrogen-fixing symbioses in legumes were known for almost a century at the time (47), as were nitrogen-fixing symbioses in single-celled marine plankton (48). Nitrogen-fixing symbionts in marine wood-boring bivalves had just been discovered (49). Although nitrogen in the form of nitrate and ammonium can be relatively abundant at the hydrothermal vents where chemosynthetic symbioses were first discovered and studied, it was unclear whether this could sustain the nitrogen requirements for these fast-growing chemosynthesis-based ecosystems. To build biomass, approximately 1 mol of nitrogen is required for every 4 to 4 mol of carbon (50, 51). Considering that the symbionts are fixing enough carbon to sustain both themselves and their hosts, some of which grow to 2 m in height, their nitrogen requirement must also be vastly higher than for chemolithoautotrophs thriving alone in vent environments. Nevertheless, early investigations showed no evidence for nitrogen-fixing activity in chemosynthetic symbionts, and the focus shifted toward other nitrogen sources that can be plentiful in these environments and could be shown to provide nitrogen in chemosynthetic symbioses (see, for example, reference 52; for a review, see reference 53). These include nitrate, ammonium, and free amino acids. Nitrate concentrations in deep-sea habitats are typically around 40 µM, and ammonium can reach millimolar concentrations (52, 54–57). The question of nitrogen fixation by marine chemosynthetic symbionts was not revisited until decades later. In the meantime, in terrestrial cave habitats, evidence emerged that the chemosynthetic *Thiothrix* symbionts of *Niphargus* amphipods may be capable of nitrogen fixation (58; see also references 59–63) for additional reviews of nitrogen-fixing symbioses.

Surprisingly, chemosynthetic symbioses were discovered at remote locations in the deep sea before we realized that they are widespread in much more accessible (and more intensively studied) shallow marine habitats that can be reached with SCUBA or snorkel rather than the oceangoing vessels and large robotic instruments needed for deep-sea exploration (2, 12). Coastal seagrass beds are a common habitat for chemosynthetic symbioses, although in contrast to hydrothermal vents, which are powered by chemosynthesis alone, in seagrass beds, photosynthesis and chemosynthesis both contribute to productivity (64). The key nutritional requirements for chemosynthetic symbioses can be found in seagrass sediments: hydrogen sulfide, produced by active sulfate-reducing bacteria in anoxic zones of marine sand, and oxygen from the overlying seawater (65). However, there is another stark contrast between seagrass sediments and deep-sea hydrothermal vents as habitats for chemosynthetic symbioses: the availability of bioavailable nitrogen for biosynthesis. Seagrasses grow best in clear oligotrophic waters which by definition have few available nutrients and are thus typically nitrogen limited (66). Accordingly, the concentration of dissolved nitrogen in seagrass sediments is typically lower than in the deep sea. Although these concentrations vary greatly in time and space, nitrate concentrations of <10 µM and ammonium concentrations of <2 µM are typical of tropical seagrass sediments (see reference 21 and references therein). Just like the waters surrounding coral reefs (which can also host chemosynthetic symbioses), these seagrass habitats are the ocean’s closest answer to the deserts found on land. In contrast to land plants, seagrasses have a more limited capacity to store nutrients (67). Thanks to these exceptional environmental challenges, seagrass growth is typically limited by available nitrogen, rather than other nutrients or trace elements, although nitrogen-fixing bacteria that might help alleviate nitrogen limitation can be found in some seagrass-associated niches (68–72).

Seagrass beds are a common habitat for burrowing clams of the family Lucinidae. Lucinids can promote seagrass health through a “nested” association between seagrasses and the lucinid bivalves and their intracellular sulfide-oxidizing symbionts (see below). Experimental evidence showed that both lucinids and seagrasses grow better together than apart (73). This beneficial association is also evident in the fossil records of seagrasses and lucinids, which indicate concurrent patterns of diversification during the Cretaceous period (74). One possible basis of this tripartite association is the removal of sulfide, a phytotoxin, from the surrounding sediments by the symbionts of lucinid bivalves; without this, the release of photosynthates from seagrass roots would lead to sulfide accumulation since it provides energy for sulfate-reducing bacteria in the anoxic zones of the seagrass “rhizosphere” and surrounding sediments. The enormous abundance of lucinid bivalves in some seagrass habitats and their prolific productivity in nitrogen-limited habitats raise the question whether they might experience nitrogen limitation and, if so, how they deal with this challenge. Two recent publications provided a possible answer by revealing that lucinid symbionts, in contrast to all other chemosynthetic symbionts investigated up until that point, encode and express a complete nitrogen fixation pathway (20, 21). The initial hypothesis that symbionts can fix nitrogen was therefore supported, it just took a few decades to finally be substantiated.

Nitrogen fixation ability was discovered concurrently in the symbionts of two lucinid species: *Loripes orbiculatus* from the Mediterranean and *Codakia orbicularis* from the Caribbean (29, 30). In sum, these two reports provided a range of evidence supporting nitrogen-fixing activity of the symbionts. This included transcriptomics and proteomics showing expression of the nitrogenase, the enzyme that catalyzes the reduction of molecular nitrogen, and associated genes for maturation and activity of nitrogenase. Nitrogen fixation genes including the nitrogenase were among the most highly expressed symbiont genes in some host individuals. Acetylene reduction assays, which detect the activity of the nitrogenase, further supported nitrogen-fixing activity by lucinid symbionts. Finally, natural stable nitrogen isotope ratios were consistent with biological nitrogen fixation directly providing a source of nitrogen for the symbiosis.

## HOW WIDESPREAD IS NITROGEN FIXATION IN CHEMOSYNTHETIC SYMBIONTS?

**FIG 1:**
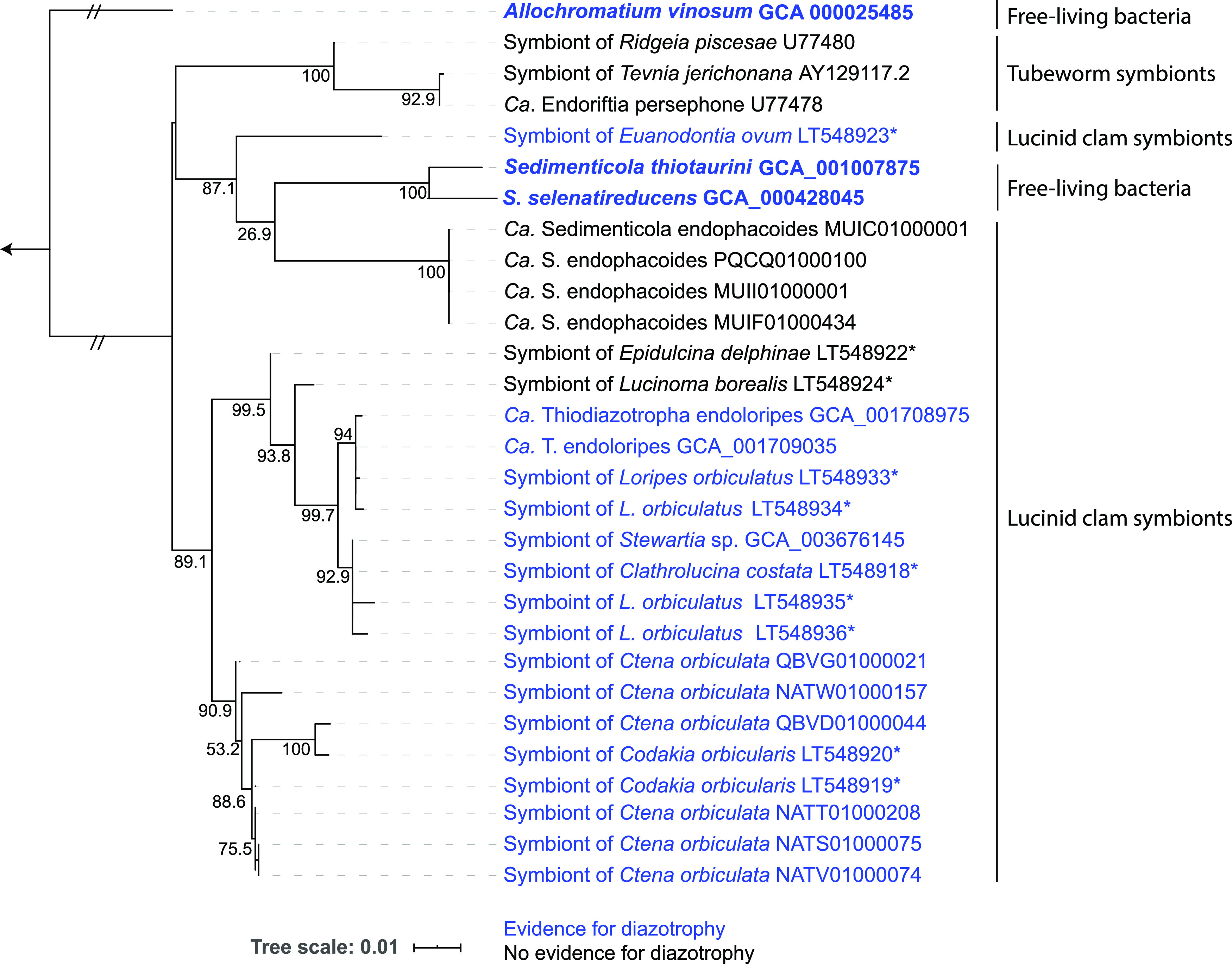
Not all lucinid symbionts are capable of nitrogen fixation and may have lost this ability on multiple occasions throughout evolution. A maximum-likelihood phylogenetic tree of lucinid symbionts and their free-living and symbiotic relatives, based on 16S rRNA genes, is shown. Symbionts capable of diazotrophy are shown in blue; those with no evidence for diazotrophic ability are shown in in black. Asterisks denote organisms for which the evidence of diazotrophy comes from PCR amplification and sequencing of the *nifH* gene (or in the case of the symbionts of *Epidulcina delphinae* and *Lucinoma borealis*, lack of PCR amplification with *nifH*-specific primers). All others are based on screening draft genome sequences for *nifH* and associated diazotrophy genes. Free-living bacteria are indicated in boldface. The tree was calculated in IQ-TREE using the TIM+F+I+G4 nucleotide substitution model (119). The internal nodes show approximate likelihood-ratio test (aLRT) SH-like support values calculated from 10,000 bootstrap replicates.

So far, eight lucinid species have been surveyed for nitrogen fixation genes, either by PCR of the diagnostic gene *nifH*, or by metagenome sequencing (Fig. 1). Intriguingly, nitrogen fixation may not be conserved among all lucinid symbionts. The *nifH* gene could not be amplified from two of the species surveyed: *Epidulcina* cf. *delphinae* from muddy sediments in Madagascar and *Lucinoma borealis* from bare sediments with no visible plant growth in Sweden (20). Although lack of a PCR product is not definitive proof that the genes are missing, *nif* genes were also missing from metagenome-assembled symbiont draft genomes from another host species, *Phacoides pectinatus*, from mangrove sediments in Florida (37). These genomes are not yet closed; thus, the *nif* genes may have been missed when binning the symbiont genome fragments from gill metagenomes. However, if these genes are truly absent, then there are two possible explanations: (i) either the common free-living ancestor of lucinid (and *Riftia*) symbionts was capable of nitrogen fixation, and this was lost independently on multiple occasions, or (ii) these genes have been gained multiple times independently by horizontal gene transfer. Considering that the closest free-living relative, Sedimenticola thiotaurini has *nif* genes (75), the first explanation may be correct. Broader sampling of symbiont genomic diversity and comparison of species phylogenies with *nif* gene phylogenies would help to distinguish these two possibilities. Presumably, the selective pressure to keep the nitrogen-fixing ability would be strongest in environments where nitrogen is most limited, and nitrogen limitation is typically more severe in shallow waters compared to the deep-sea habitats colonized by chemosynthetic symbioses. However, the picture is probably more complex. For example, fiddler crabs from mangrove habitats were recently shown to be “hot spots” of microbial symbiotic nitrogen fixation (76). This contrasts with the observation that the symbionts from mangrove-associated lucinids do not have the ability to fix nitrogen.

Beyond lucinid clams, *nif* genes were also found in the genomes of sulfide-oxidizing ectosymbionts from marine nematode worms (20). These symbionts are not the closest relatives of the lucinid symbionts; thus, nitrogen-fixing chemosynthetic symbionts can be found in multiple bacterial groups and in multiple host animal groups. Here, the environment seems to be the strongest driver of symbiont metabolic capability, since these worms also inhabit nutrient-poor oligotrophic reef sands.

## ECOLOGICAL IMPACTS OF NITROGEN FIXATION BY CHEMOSYNTHETIC SYMBIONTS: HOST-MICROBE-ENVIRONMENT INTERACTIONS

Considering that each lucinid clam hosts a massive population of symbionts and that these clams can reach densities of thousands of individuals per m^2^ of sediment, they have remarkable potential as a source of bioavailable nitrogen to oligotrophic coastal ecosystems (77). A few studies have revealed potential impacts of diazotrophic chemosynthetic symbionts on nitrogen budgets in seagrass sediments. In fact, the first indications that these symbioses affect nitrogen cycling came from measurements of nutrient availability in lucinid habitats long before it became clear that the symbionts are capable of diazotrophy. Reynolds et al. (65) showed that seagrass sediments with lucinids typically had higher ammonium concentrations than sediments without lucinids. This effect was considered small compared to other sediment-dwelling mussels that are not known to host chemosynthetic symbionts and whose filter-feeding activity can double available ammonium in seagrass sediments (78, 79). However, this ammonium comes from remineralization of feces and pseudofeces deposited by the bivalves in the sediment. This “biodeposition” brings organic material produced via photosynthesis in the overlying water column, and filtered by the bivalves, into the sediment. In contrast to the mytilid mussels in these experiments, lucinid clams have a reduced capacity to filter feed thanks to their long history of reliance on their gill symbionts for nutrition. This means that the additional ammonium made available by lucinids is more likely due to symbiont nitrogen fixation. At the ecosystem level, biodeposition simply results in a reshuffling of bioavailable nitrogen between water column and sediment compartments, while lucinids may provide a net source of new nitrogen to seagrass ecosystems. Recent experimental evidence supported this view by showing net excretion of ammonium by diazotrophic symbiont-hosting lucinids in incubations without experimental addition of particulate organic matter (51). The method used to quantify ammonium fluxes in this study, isotope pool dilution, is well established in terrestrial ecology but has not yet been widely applied in marine ecology. This is surprising because it has the great advantage that it quantifies turnover rates, essential in habitats where production and consumption occur simultaneously. The ammonium release measured in symbiont-containing lucinids was up to 10 times higher than rates measured in bivalves that do not host chemosynthetic, diazotrophic symbionts (51).

The Cardini et al. (51) study also highlights the impact of seasons on the physiology of hosts and symbionts, since both carbon and nitrogen fixation rates were higher in the fall than in the spring, the only two seasons tested. Carbon fixation rates had the largest differences and were 10 times higher in the fall than in the spring. Seasonal differences in reliance on symbiotic nutrition were hinted at previously by a natural stable carbon isotope modeling study, which predicted a greater reliance on filter-feeding particulate organic matter from June to January by lucinids in seagrass meadows off the coast of Africa (77). Intriguingly, this coincides with the period when these bivalves produce gametes. van der Geest et al. hypothesized that nutritional supplementation by filter-feeding is particularly important to provide additional energy for reproduction in this lucinid species (77). A histological study on the same lucinid species from a different location showed a striking correlation between symbiont abundance and the reproductive cycles of the host, with symbiont biomass substantially lower during gamete development (21). These bivalves were hypothesized to “mobilize” symbiont organic matter to provide energy for reproduction.

Another intriguing link between lucinids and their surrounding environment was recently uncovered through worldwide molecular surveys and imaging of the microbial diversity in the rhizosphere of a range of seagrasses (80). This revealed that relatives of lucinid symbionts, which fall within the “*Candidatus* Thiodiazotropha” genus are widespread members of the seagrass rhizosphere microbiome. Currently, the only whole-genome data available for this genus come from lucinid symbionts; no free-living or plant-associated “*Ca*. Thiodiazotropha” genome data are available to search for nitrogenase-encoding genes, but *nifH* genes amplified and sequenced from seagrass sediments cluster together with those from lucinid symbionts, indicating that these free-living relatives may also be capable of nitrogen fixation (21). It will be intriguing to see whether these are actively fixing nitrogen in the sediment and seagrass rhizosphere, and if so, whether symbiotic or free-living “*Ca*. Thiodiazotropha” contribute more to the nitrogen budget of seagrass ecosystems.

Clearly, seasonal changes in the environment drive both host and symbiont physiology. Key environmental factors that could influence symbiont activity, particularly nitrogen fixation include seagrass growth and dormancy, temperature-dependent differences in surrounding sulfate reduction rates, which drive sulfide provision to power symbiont metabolism, and the activity of nitrogen-fixing microorganisms in diverse surrounding habitats from sediments to the water column to the seagrass rhizosphere. This should be considered when planning and interpreting incubation experiments to test symbiont functions, including nitrogen fixation, since symbiont metabolic function may shift across seasons. This variability poses an enormous challenge for experiments to quantify symbiont function. However, it is also a unique opportunity to tease apart host-microbe-environment interactions in nature and to understand how holobionts respond to environmental change. Unraveling the factors responsible will require us to extend the current irregular opportunity-based field campaigns typical of much marine research to longer-term time series studies. Just as symbionts can be both advantageous and detrimental depending on the life history stage of the host (for example, in corals), the functions and benefits of chemosynthetic symbionts may shift with changing seasons (6, 81).

## REMAINING QUESTIONS

One of the most puzzling observations from Petersen et al. (20) was the difference in *nif* gene expression between the symbiont populations of different clam individuals. At the transcriptome level, *nif* genes were some of the most highly expressed, as high as those involved in sulfide oxidation, but only in two of five individuals sequenced. In the other three, *nif* genes were barely or undetectable in the metatranscriptomes (20). In addition, six individuals were analyzed with shotgun proteomics. Nif proteins were detected in five of these individuals, but not in the sixth individual. All clam individuals for each analysis type were sampled from the same location at the same time on the same day. All were of a similar size and were collected from approximately the same sediment depth. For a start, this indicates that nitrogen fixation is differentially regulated in these symbionts, in contrast to other core functions such as carbon fixation and sulfur oxidation, which are without exception the most abundantly expressed symbiont pathways in every host individual tested so far. If regulation of nitrogen fixation expression is driven by environmental factors such as availability of nitrogen nutrients, then the availability of these nutrients must be patchy on an extremely fine scale. Patchiness at the centimeter scale in seagrass sediments is not unprecedented (66). Moreover, in lucinid habitats in the Mediterranean Sea, we often observe “pockets” of seagrass debris buried in the sediments (unpublished data). If this debris is powering sulfate reduction and the concurrent production of sulfide in the sediments, then this could explain such patchiness in the environment, but so far, debris, sulfide production, and nitrogen availability have not been quantified extensively at this scale. High-resolution *in situ* mapping of the biological and chemical landscape of seagrass sediments over time and space will provide more answers, as would aquarium experiments to manipulate environmental parameters such as nitrogen, oxygen, and sulfide availability. Because the host can massively influence the microenvironment of the symbionts through valve opening and closing or through its burrowing and water pumping activity, it may also be informative to do such activity measurements on live symbiont cells extracted from host tissues until pure cultures are available to circumvent the confounding influence of host behavior.

**FIG 2:**
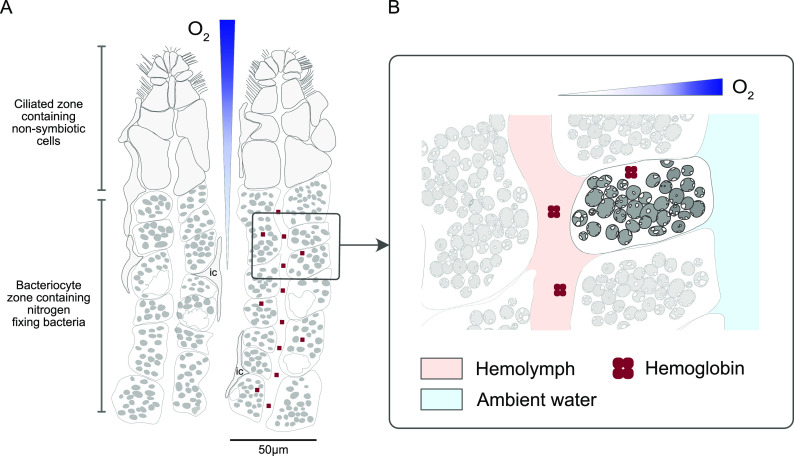
Symbionts may be supplied with oxygen by diffusion from ambient seawater and host hemoglobins. This image is a model of hypothesized oxygen delivery systems to the symbionts of lucinid clams. (A) The image on the left shows two gill filaments, which are made up of a single layer of epithelial cells surrounding a lumen of circulatory fluid, the hemolymph. This epithelium is made up of symbiont-containing bacteriocyte cells, and symbiont-free cells with other functions (shaded in gray) such as ciliated cells at the outer edge, and intercalary cells (ic) that partially cover the bacteriocytes. Oxygen is expected to be depleted away from the ambient seawater (fully oxygenated) which first flows over the ciliated edges of the gill filaments (gradient shown in panel A). (B) Oxygen may also become depleted toward the hemolymph-facing side of the bacteriocytes as oxygen diffuses from the ambient seawater into the bacteriocytes and is consumed by the symbionts. Functional oxygen-binding hemoglobins have been found in lucinid hemolymph, and they also express intracellular hemoglobins. Thus far, this proposed model has not been experimentally tested.

Nitrogenase is an oxygen-sensitive enzyme, and throughout nature an impressive range of adaptations have emerged to protect it from oxygen. On the face of it, it might seem illogical to host a population of nitrogen-fixing symbionts in the gill, the site of gas exchange in a range of marine animals. However, in bivalves, this gas exchange function may be carried out in other tissues, as the gill is primarily used for filter feeding (32, 53). Nevertheless, a bivalve gill is a well-ventilated surface, and the symbionts colonize the apical edges of gill cells, closest to the surrounding seawater, and are thus directly exposed to dissolved gases in seawater, including oxygen, that can diffuse into gill tissues (Fig. 2). Rates of diffusion across the gill surface, as well as consumption by the host and its symbionts, are not well understood, but considering the intense oxygen demand of sulfide oxidation, symbionts may experience steep microgradients of oxygen availability, with those at the apical bacteriocyte edge having substantially more access to oxygen than those toward the basal edge closest to the hemolymph, the animal’s circulatory fluid. Such fine-scale gradients would be challenging to measure, but if they do exist, they could result in spatial structuring of phenotypically distinct symbiont subpopulations. For example, symbiont cells located further away from the apical edge, exposed to less oxygen, may inhabit a microhabitat ideal for nitrogen fixation. In such a microhabitat, the capacity to use alternative electron acceptors when oxygen is limiting would provide a selective advantage, and accordingly, the genomes encode the potential to respire alternative electron acceptors such as nitrate (20, 21, 37). Moreover, activity measurements have shown that symbiont populations respire oxygen and nitrate simultaneously (82, 83). They also encode a highly expressed terminal oxidase annotated as a DMSO reductase, although the substrate of this enzyme has not been experimentally verified (20). Counter to expectations, a few studies have shown that nitrogen fixation increases with oxygen availability. In plants, high oxygen concentrations around nodules transiently increase nitrogenase activity (84). The chemosynthetic nitrogen-fixing ectosymbionts of marine nematode worms incubated under oxic conditions expressed significantly more nitrogenase than those incubated under anoxic conditions (85). Although the reasons for this are not yet fully understood, one theory is that oxygen respiration is needed to provide enough energy to power the metabolically “expensive” process of nitrogen fixation. Finally, hosts might also influence oxygen concentrations at a fine scale by binding oxygen with intracellular hemoglobins (see more on lucinid hemoglobins below).

If genetically homogenous but physiologically distinct symbiont subpopulations inhabit different regions of the gill or bacteriocytes, they may have different functions in the symbiosis; it is conceivable that the “labor” of symbiosis is divided into a subpopulation that provides carbon and another one that provides nitrogen. Having distinct cells “tuned” to a particular metabolic task can enhance the efficiency of a genetically homogenous population (86). Partitioning of nitrogen and carbon fixation into distinct cells is seen in some multicellular cyanobacteria, although restricting nitrogen fixation to dedicated heterocyst cells is primarily thought to protect nitrogenase from the oxygen produced through photosynthesis, rather than the ambient environment as we propose in the lucinid symbionts (87).

Metabolic partitioning in subpopulations is invisible with most “omics” methods, although physical separation of morphologically distinct subpopulations can help to overcome this problem (24). Methods for measuring microbial activity that preserve spatial structure such as mRNA-FISH, HCR-FISH, immunohistochemistry, and nanoSIMS would be ideal for testing the theory of phenotypically distinct symbiont subpopulations. They should ideally be combined, since nitrogen-fixing cells may pass fixed nitrogen compounds directly to nonfixing neighbors, introducing a “cross-feeding” artifact in isotope labeling experiments. In nitrogen-fixing cyanobacteria, redistribution of newly fixed nitrogen can happen within minutes (88). Indeed, problematically, cells with the highest carbon fixation rates may have the highest nitrogen demand if fixed carbon is used for the biosynthesis of N-containing compounds rather than directly released as carbohydrates for host nutrition. Lucinid symbionts of the same genotype can display distinct morphologies, ranging from <1 to ∼5 μm in diameter. This is intriguingly reminiscent of the morphological diversity of some plant nodule symbionts. If like plant nodule symbioses, these different morphologies reflect phenotypic differences that result in some cells specializing in, e.g., nitrogen fixation (89), this would be a striking example of convergent evolution in nitrogen-fixing symbioses in plants and animals.

## ESTABLISHING AND MAINTAINING NITROGEN-FIXING ENDOSYMBIOSES IN PLANT AND ANIMAL HOSTS: COMMON CHALLENGES, COMMON SOLUTIONS?

The discovery that marine lucinid symbionts fix nitrogen is an inspiration to compare these with the intensively studied root nodule rhizobial symbioses on land, where nitrogen provision is at the heart of these widespread and ancient associations. Comparing them could help us learn something new about each system or even about general principles of biological nitrogen fixation or intracellular symbiosis. We acknowledge that diazotrophic symbioses in plants are functionally and phylogenetically diverse, and that not all of the mechanisms described here apply in all plant-microbe symbioses. Moreover, a comparison with other marine nitrogen-fixing symbioses such as those between unicellular algae and nitrogen-fixing bacteria would also be intriguing (62, 90) but is beyond the scope of this review, which focuses on comparing lucinid symbioses with some intracellular plant nodule symbioses.

Numerous challenges unite the seemingly disparate nitrogen-fixing associations in multicellular hosts in the sea and on land. To start with, in both, symbionts are recruited from the environment during host development and must be attracted to the site of colonization, recognized, and then internalized. We know vastly more about these processes in some plant symbioses than in lucinid clams. Sequences identical to the lucinid symbionts are rarely, if ever detected in their surrounding environments (91; our unpublished analyses), and although symbiont-free juveniles can be reared in the lab and symbiosis induced experimentally (41), there is currently nothing known about the cues that draw in symbionts from the environment, or the recognition mechanisms that determine specificity and allow their internalization. Plants exude polyphenolic compounds, flavonoids, into their immediate surroundings to attract rhizobia from the soil. Flavonoids are only known to be produced by plants and fungi. The most conspicuous organic substance released by marine bivalves is mucus. Mucus is a remarkably versatile substrate that coats exterior surfaces and is used for diverse functions such as trapping and transporting food and reinforcing burrow walls (92, 93). Mucus plays a key role in immune interactions, traditionally studied in pathogenesis, and more recently also in beneficial interactions (94). In the well-studied marine squid-bioluminescent *Vibrio* symbiosis, the squid mucus is a crucial site for microbial sorting, symbiont attraction, and recognition (95, 96). In the marine stilbonematinid nematode ectosymbionts that also potentially fix nitrogen, a C-type lectin in the mucus surrounding the animal mediates recognition and attachment (97). Bivalve mucus can stimulate the growth of their free-living photosynthetic microalgal food sources, even though the main energy source for these algae is sunlight (98). Lucinids are prolific mucus producers and could potentially structure the surrounding sediment microbial communities through burrow construction and mucus secretion. In other animal-microbe associations, the burrow is used to “farm” bacteria that are used for animal food in the “traditional” sense of ingestion by the mouth (99). Lucinids may have evolved a similar but nevertheless unique strategy of using their burrows to attract symbionts, perhaps farming them in the environment and within their own bodies before digesting them intracellularly to gain nutrition.

Once intracellular symbiosis has been established, controlling symbiont growth becomes a major challenge, one that is shared by both plant and animal hosts. The model legume Medicago truncatula inhibits symbiotic cell division, leading to polyploidy of the symbiont cells within nodules. *M. truncatula* encodes hundreds of distinct nodule-specific cysteine-rich proteins, termed NCRs, in its genome (100, 101). One of these, NCR247, enters the bacterial cytosol and binds to FtsZ, one of the key proteins orchestrating bacterial cell division (102). NCR247 prevents symbiont cell division and induces polyploidy and cell elongation. Like the rhizobial symbionts of *M. truncatula*, lucinid symbionts also show extensive morphological diversity, with large, elongated cells and are also polyploid (103). This raises the intriguing prospect that both legumes and lucinid clams have convergently evolved a similar strategy to control their intracellular symbionts’ growth. Antimicrobial peptides (AMPs) such as NCRs also seem to play similar roles in insect symbioses. For example, a single AMP induces similar effects, including inhibition of cell division and symbiont cell elongation in the weevil *Sitophilus* (104). Although the mechanism of cell division inhibition is completely unknown in the lucinids, it is difficult to say what would be the more surprising outcome: (i) that clams, insects, and legumes evolved the same mechanism to control intracellular symbiont growth independently or (ii) that multiple, distinct systems have evolved to solve the same biological problem. Either way, this is clearly an exciting area for future research. As Maróti and Kondorosi pointed out in an excellent review in 2014, polyploidy and inhibition of cell division by host-derived peptides may indeed be general principles underpinning several intracellular symbioses (105).

Another aspect of controlling symbiont activity, common to animal and plant symbioses is providing (or limiting) oxygen flux to intracellular symbionts. Leguminous plants use intracellular oxygen-binding proteins, called leghemoglobins, to control oxygen flux to the symbionts. Animals that host chemosynthetic symbionts also make hemoglobin proteins to bind oxygen. Lucinid clams in particular encode multiple distinct hemoglobins to bind either sulfide or oxygen (106–108). Their expression can be restricted to the symbiont-hosting gill tissues, and they are localized to the bacteriocytes (109, 110). This raises the possibility that the lucinid hemoglobins may perform a similar function to the leghemoglobins in controlling oxygen concentrations in the symbionts’ immediate habitat, as suggested by Dando et al. (111). In addition to leghemoglobins, a structural feature, the cortical diffusion barrier, restricts oxygen exposure in root nodules (112). It is not known whether lucinids have a similar feature, but their gills are coated with mucus, which has been shown to limit oxygen diffusion (113–115).

**FIG 3:**
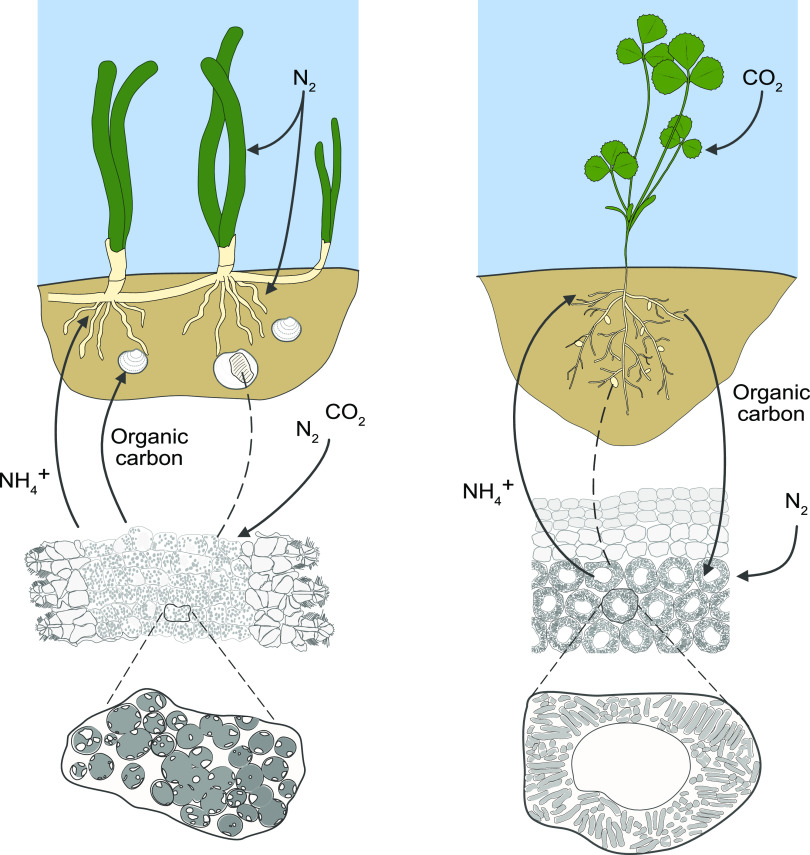
Comparing carbon and nitrogen fluxes in intracellular nitrogen-fixing symbioses on land and in the sea using lucinid clams (left) and Medicago truncatula (right) as examples. Bacterial cells are housed in specialized host cells in both systems. Bacterial symbiont cells are polyploid and show morphological heterogeneity in both systems. The major difference is that in the *M*. *truncatula* root nodule example, the host provides organic carbon to the rhizobia symbionts and gains fixed nitrogen in exchange (arrows show direction of nutrient transfer, from host above to symbionts below). In the lucinid symbiosis, the bacterial symbionts provide organic carbon, and possibly also fixed nitrogen to the host, which provides a source of nitrogen to the seagrass sediments the hosts inhabit. Although not shown here, nitrogen fixation can occur in other seagrass-associated niches (see the main text).

One major difference between diazotrophic symbioses in plants and in lucinid clams is the direction of carbon flow (Fig. 3). Plant symbioses function as a nutritional exchange. The host provides all of the symbionts’ carbon, in exchange for nitrogen, but in lucinids, do the symbionts provide both? This would be surprising in comparison to plant symbioses, where the fixed carbon the symbionts gain from the host is the major advantage of the association, and is the reason why they fix and share nitrogen. Numerous studies have demonstrated carbon fixation by lucinid symbionts (see, for example, references 51, 111, and 116). Despite this, their genomes encode pathways for heterotrophy, which are expressed by the symbionts within the host (20, 21). Potentially, they could also take up carbon compounds from their hosts. In other chemosynthetic symbioses, the host is thought to provide metabolic intermediates that the symbionts cannot synthesize by themselves (22). This explains why chemoautotrophic symbionts encode and express transporters for organic compounds: they are thought to take these up to compensate for incomplete metabolic pathways, rather than using them as a source of carbon. However, lucinid symbiont genomes seem to encode complete pathways for central carbon metabolism; therefore, the question remains why they would use a mixotrophic strategy within their host, whether the hosts provide them with particular carbon substrates, and if so, under what conditions. Intriguingly, their closest cultured relative, Sedimenticola thiotaurini, also a diazotrophic sulfide-oxidizing chemolithoautotroph, is more tolerant to oxygen when supplemented with organic carbon (117). From the lucinid host’s perspective, providing the symbionts with organic carbon would also be an opportunity to modulate the symbionts’ metabolism to match their own nutritional needs. For example, providing organic carbon substrates would shift the C/N balance of the symbionts, possibly promoting symbiont nitrogen fixation to compensate, as shown previously in other diazotrophs such as Rhodobacter capsulatus (118).

The all-rounder in sport is a flexible player capable of carrying out multiple functions or of playing multiple roles or positions equally well, but the all-rounder is almost by definition not the best performer in any of these roles. Flexibility comes at the cost of peak performance. Does this apply to chemosynthetic symbiotic all-rounders? If they are routing metabolic energy gained from sulfur oxidation to nitrogen fixation, then this must reduce their maximum capacity to fix carbon. Future efforts to quantify symbiont activity at the single-cell level will tell us whether the symbiont population of a single host animal is a team of genetically identical individuals divided up into metabolic specialists, and if so, how these fit together to form a functioning symbiotic partnership.
